# Supplementary material for: Patient Characteristics Associated with Growth of Patient-Derived Tumor Implants in Mice (Patient-Derived Xenografts)
Source: Cancers (Basel). 2023 Nov 14;15(22):5402. doi: 10.3390/cancers15225402 (PMC10670531; doi:10.3390/cancers15225402)
Supplement: Supplementary file 1 [file cancers-15-05402-s001.zip › cancers-2639339-supplementary.pdf]

SUPPLEMENTARY DATA

Table S1: ROC Data For Tumor Growth Rates in the first and second passages. Specificity and Sensitivity for predicting final engraftment success.

Threshold	Sensitivity	Specificity	Positive Predictive Value	Negative Predictive Value
**-93.2**	100.0	1.4	72.2	100.0
**-92.3**	99.5	1.4	72.1	50.0
**-90**	99.5	2.7	72.4	66.7
**-88**	99.5	4.1	72.7	75.0
**-87**	99.5	5.5	72.9	80.0
**-85.7**	99.5	6.8	73.2	83.3
**-85.2**	99.5	8.2	73.5	85.7
**-83.9**	99.5	9.6	73.8	87.5
**-82.5**	98.9	9.6	73.7	77.8
**-81.4**	98.4	9.6	73.6	70.0
**-80.2**	98.4	11.0	73.9	72.7
**-79.8**	98.4	12.3	74.2	75.0
**-78.3**	98.4	13.7	74.5	76.9
**-75.8**	98.4	15.1	74.8	78.6
**-74**	98.4	16.4	75.1	80.0
**-71.6**	98.4	17.8	75.4	81.2
**-69**	98.4	19.2	75.7	82.4
**-67.3**	98.4	20.5	76.0	83.3
**-66.4**	98.4	21.9	76.3	84.2
**-66.2**	98.4	23.3	76.7	85.0
**-65.6**	97.9	23.3	76.6	81.0
**-64.2**	97.3	23.3	76.5	77.3
**-60.2**	97.3	24.7	76.8	78.3
**-56.9**	96.8	24.7	76.7	75.0
**-55.7**	96.3	24.7	76.6	72.0
**-54.8**	96.3	26.0	76.9	73.1
**-54.2**	96.3	27.4	77.3	74.1
**-53.5**	96.3	28.8	77.6	75.0
**-53**	96.3	30.1	77.9	75.9
**-52.4**	95.7	30.1	77.8	73.3
**-45.3**	95.2	30.1	77.7	71.0
**-38**	94.7	30.1	77.6	68.8
**-36.3**	94.1	30.1	77.5	66.7
**-32.2**	93.6	30.1	77.4	64.7
**-25.3**	93.6	31.5	77.8	65.7
**-20.8**	93.0	31.5	77.7	63.9
**-20.2**	93.0	32.9	78.0	64.9
**-18.3**	92.5	32.9	77.9	63.2
**-14**	92.5	34.2	78.3	64.1
**-10.4**	92.5	35.6	78.6	65.0
**-9.3**	92.5	37.0	79.0	65.9
**-8.9**	92.5	38.4	79.4	66.7
**-6.8**	92.0	38.4	79.3	65.1
**-3**	92.0	39.7	79.6	65.9
**1.3**	92.0	41.1	80.0	66.7
**6.1**	90.9	41.1	79.8	63.8
**10.5**	90.9	42.5	80.2	64.6

**12.6**	90.9	43.8	80.6	65.3
**12.9**	90.4	43.8	80.5	64.0
**13.8**	89.8	43.8	80.4	62.7
**15.1**	89.8	45.2	80.8	63.5
**18.4**	89.3	45.2	80.7	62.3
**22**	88.8	45.2	80.6	61.1
**26**	88.8	46.6	81.0	61.8
**29.5**	88.8	47.9	81.4	62.5
**32.6**	88.2	47.9	81.3	61.4
**36**	87.7	47.9	81.2	60.3
**41.1**	87.2	47.9	81.1	59.3
**46.3**	86.6	47.9	81.0	58.3
**47.7**	86.6	49.3	81.4	59.0
**48.3**	86.1	49.3	81.3	58.1
**49.6**	86.1	50.7	81.7	58.7
**50.9**	85.6	50.7	81.6	57.8
**54.7**	85.0	50.7	81.5	56.9
**58.9**	85.0	52.1	82.0	57.6
**60**	84.5	52.1	81.9	56.7
**62.1**	84.0	52.1	81.8	55.9
**64.4**	84.0	53.4	82.2	56.5
**65.7**	83.4	53.4	82.1	55.7
**66.4**	83.4	54.8	82.5	56.3
**67.4**	82.9	54.8	82.4	55.6
**68.7**	82.4	54.8	82.4	54.8
**69.8**	82.4	56.2	82.8	55.4
**70.9**	82.4	57.5	83.2	56.0

72.8	82.4	58.9	83.7	56.6
**74.2**	81.8	58.9	83.6	55.8
**75.7**	81.3	58.9	83.5	55.1
**77.1**	80.7	58.9	83.4	54.4
**77.5**	80.2	58.9	83.3	53.8
**80.6**	79.7	58.9	83.2	53.1
**87.4**	79.1	58.9	83.1	52.4
**92.6**	79.1	60.3	83.6	53.0
**96.1**	78.6	60.3	83.5	52.4
**98.5**	78.1	60.3	83.4	51.8
**100.4**	77.5	60.3	83.3	51.2
**102.6**	77.5	61.6	83.8	51.7
**104.2**	77.0	61.6	83.7	51.1
**106.5**	76.5	61.6	83.6	50.6
**108.3**	75.9	61.6	83.5	50.0
**109.8**	75.4	61.6	83.4	49.5
**113.1**	75.4	63.0	83.9	50.0
**119.2**	75.4	64.4	84.4	50.5
**125.5**	74.9	64.4	84.3	50.0
**130.4**	74.3	64.4	84.2	49.5
**133.1**	73.8	64.4	84.1	49.0
**134.5**	73.3	64.4	84.0	48.5
**135.7**	72.7	64.4	84.0	48.0
**136.9**	72.7	65.8	84.5	48.5
**138.6**	72.2	65.8	84.4	48.0
**140.1**	71.7	65.8	84.3	47.5
**142.1**	71.1	65.8	84.2	47.1
**147.2**	70.6	65.8	84.1	46.6
**151.3**	70.6	67.1	84.6	47.1
**152.9**	70.1	67.1	84.5	46.7
**155.5**	69.5	67.1	84.4	46.2
**159**	69.0	67.1	84.3	45.8
**162.3**	68.4	67.1	84.2	45.4
**163.8**	67.9	67.1	84.1	45.0
**165.2**	67.4	67.1	84.0	44.5
**170**	66.8	67.1	83.9	44.1
**175**	66.3	67.1	83.8	43.8
**176.8**	65.8	67.1	83.7	43.4
**180.5**	65.8	68.5	84.2	43.9
**185.1**	65.2	68.5	84.1	43.5
**187.7**	64.7	68.5	84.0	43.1
**192.3**	64.2	68.5	83.9	42.7
**195.5**	63.6	68.5	83.8	42.4
**196**	63.1	68.5	83.7	42.0
**197**	62.6	68.5	83.6	41.7
**198.9**	62.0	68.5	83.5	41.3
**204.8**	61.5	68.5	83.3	41.0
**209.7**	61.5	69.9	83.9	41.5
**214.4**	61.0	69.9	83.8	41.1
**219.3**	60.4	69.9	83.7	40.8
**224.6**	60.4	71.2	84.3	41.3
**229.9**	59.9	71.2	84.2	40.9
**230.8**	59.9	72.6	84.8	41.4
**233.2**	59.4	72.6	84.7	41.1

**237.3**	58.8	72.6	84.6	40.8
**240.2**	58.3	72.6	84.5	40.5
**248.1**	57.8	72.6	84.4	40.2
**256.3**	57.2	72.6	84.3	39.8
**259.3**	56.7	72.6	84.1	39.6
**261.9**	56.1	72.6	84.0	39.3
**263.5**	55.6	72.6	83.9	39.0
**265.8**	55.1	72.6	83.7	38.7
**268**	55.1	74.0	84.4	39.1
**269.1**	54.5	74.0	84.3	38.8
**270.4**	54.0	75.3	84.9	39.0
**270.9**	53.5	75.3	84.7	38.7
**271.6**	52.9	75.3	84.6	38.5
**274.8**	52.4	75.3	84.5	38.2
**279**	52.4	76.7	85.2	38.6
**281.5**	51.9	76.7	85.1	38.4
**285.7**	51.3	76.7	85.0	38.1
**289.2**	50.8	76.7	84.8	37.8
**290.7**	50.8	78.1	85.6	38.3
**304.6**	50.3	78.1	85.5	38.0
**318.2**	49.7	78.1	85.3	37.7
**321.2**	49.2	78.1	85.2	37.5
**325.8**	48.7	78.1	85.0	37.3
**328.5**	48.1	78.1	84.9	37.0
**328.8**	47.6	78.1	84.8	36.8
**332.3**	47.1	78.1	84.6	36.5
**340**	46.5	78.1	84.5	36.3
**346.4**	46.0	78.1	84.3	36.1
**354.1**	45.5	78.1	84.2	35.8
**359.6**	44.9	78.1	84.0	35.6
**361.3**	44.4	78.1	83.8	35.4
**363.1**	43.9	78.1	83.7	35.2
**363.4**	43.3	78.1	83.5	35.0
**365.5**	42.8	78.1	83.3	34.8
**367.9**	42.8	79.5	84.2	35.2
**369.6**	42.8	80.8	85.1	35.5
**372.1**	42.2	80.8	84.9	35.3
**378.2**	41.7	80.8	84.8	35.1
**383.6**	41.2	80.8	84.6	34.9
**389.3**	40.6	80.8	84.4	34.7
**400.3**	40.6	82.2	85.4	35.1
**406.3**	40.6	83.6	86.4	35.5
**407**	40.1	83.6	86.2	35.3
**411.1**	39.6	83.6	86.0	35.1
**420.1**	39.0	83.6	85.9	34.9
**427.6**	38.5	83.6	85.7	34.7
**430.4**	38.0	83.6	85.5	34.5
**432.3**	37.4	83.6	85.4	34.3
**433.5**	36.9	83.6	85.2	34.1
**434.8**	36.4	83.6	85.0	33.9
**439**	35.8	83.6	84.8	33.7
**442.5**	35.3	83.6	84.6	33.5
**445.5**	34.8	83.6	84.4	33.3
**449.4**	34.8	84.9	85.5	33.7
**451.2**	34.2	84.9	85.3	33.5

**454.7**	33.7	84.9	85.1	33.3
**458.3**	33.2	84.9	84.9	33.2
**460.9**	32.6	84.9	84.7	33.0
**466**	32.1	84.9	84.5	32.8
**472.9**	31.6	84.9	84.3	32.6
**479.5**	31.0	84.9	84.1	32.5
**483.7**	30.5	84.9	83.8	32.3
**485.3**	29.9	84.9	83.6	32.1
**488.7**	29.4	84.9	83.3	32.0
**497.8**	28.9	84.9	83.1	31.8
**507.9**	28.3	84.9	82.8	31.6
**518.3**	27.8	84.9	82.5	31.5
**525**	27.3	84.9	82.3	31.3
**526.6**	26.7	84.9	82.0	31.2
**529.8**	26.2	84.9	81.7	31.0
**533.6**	25.7	84.9	81.4	30.8
**540**	25.1	84.9	81.0	30.7
**550.2**	25.1	86.3	82.5	31.0
**556.1**	24.6	86.3	82.1	30.9
**559.7**	24.1	86.3	81.8	30.7
**570.2**	23.5	86.3	81.5	30.6
**584.3**	23.0	86.3	81.1	30.4
**593.5**	22.5	86.3	80.8	30.3
**604.6**	21.9	86.3	80.4	30.1
**615.6**	21.4	86.3	80.0	30.0
**629.4**	20.9	86.3	79.6	29.9
**645.5**	20.3	86.3	79.2	29.7
**653.2**	19.8	86.3	78.7	29.6
**686.8**	19.3	86.3	78.3	29.4
**726.7**	18.7	86.3	77.8	29.3
**738.7**	18.2	86.3	77.3	29.2
**741**	17.6	86.3	76.7	29.0
**745.5**	17.1	86.3	76.2	28.9
**769.7**	16.6	86.3	75.6	28.8
**797**	16.6	87.7	77.5	29.1
**807**	16.0	87.7	76.9	29.0
**820.6**	16.0	89.0	78.9	29.3
**832.5**	15.5	89.0	78.4	29.1
**834.5**	15.0	89.0	77.8	29.0
**841.8**	14.4	89.0	77.1	28.9
**873.5**	13.9	89.0	76.5	28.8
**900.7**	13.9	90.4	78.8	29.1
**926.9**	13.4	90.4	78.1	28.9
**982.2**	12.8	90.4	77.4	28.8
**1018.8**	12.3	90.4	76.7	28.7
**1053.8**	11.8	90.4	75.9	28.6
**1108.7**	11.2	90.4	75.0	28.4
**1137.1**	11.2	91.8	77.8	28.8
**1142.4**	10.7	91.8	76.9	28.6
**1152.8**	10.7	93.2	80.0	28.9
**1161.8**	10.2	93.2	79.2	28.8
**1166.1**	9.6	93.2	78.3	28.7
**1204.4**	9.1	93.2	77.3	28.6
**1245.8**	8.6	93.2	76.2	28.5
**1257.9**	8.6	94.5	80.0	28.8

**1276.4**	8.0	94.5	78.9	28.6
**1309.8**	7.5	94.5	77.8	28.5
**1346.3**	7.0	94.5	76.5	28.4
**1419.1**	7.0	95.9	81.2	28.7
**1502.2**	6.4	95.9	80.0	28.6
**1539.5**	5.9	95.9	78.6	28.5
**1679.9**	5.3	95.9	76.9	28.3
**1851.4**	4.8	95.9	75.0	28.2
**1930.5**	4.3	95.9	72.7	28.1
**2001.7**	3.7	95.9	70.0	28.0
**2038**	3.2	95.9	66.7	27.9
**2122.1**	2.7	95.9	62.5	27.8
**2287.3**	2.1	95.9	57.1	27.7
**2666.1**	1.6	95.9	50.0	27.6
**3604.9**	1.6	97.3	60.0	27.8
**4638.6**	1.1	97.3	50.0	27.7
**5226.4**	1.1	98.6	66.7	28.0
**5708.3**	0.5	98.6	50.0	27.9
**10821.4**	0.5	100.0	100.0	28.2

Table S2: ROC Data For Tumor Growth Rates in the second passage. Specificity and Sensitivity for predicting final engraftment success.

Threshold	Sensitivity	Specificity	Positive Predictive Value	Negative Predictive Value
**270.9**	99.5	0.0	87.7	0.0
**287**	98.9	0.0	87.7	0.0
**381.3**	98.9	3.8	88.1	33.3
**524.5**	98.9	7.7	88.5	50.0
**612**	98.9	11.5	88.9	60.0
**688**	98.9	15.4	89.4	66.7
**760.5**	98.4	15.4	89.3	57.1
**882.9**	97.9	15.4	89.3	50.0
**985.6**	97.3	15.4	89.2	44.4
**1002.2**	97.3	19.2	89.7	50.0
**1074**	96.8	19.2	89.6	45.5
**1260.7**	96.8	23.1	90.0	50.0
**1412**	96.3	23.1	90.0	46.2
**1634.7**	96.3	26.9	90.5	50.0
**1867.8**	96.3	30.8	90.9	53.3
**1911.1**	95.7	30.8	90.9	50.0
**1921.3**	95.2	30.8	90.8	47.1
**2003.2**	94.7	30.8	90.8	44.4
**2100.8**	94.1	30.8	90.7	42.1
**2247**	93.6	30.8	90.7	40.0
**2402**	93.0	30.8	90.6	38.1
**2437.8**	92.5	30.8	90.6	36.4
**2475**	92.5	34.6	91.1	39.1
**2521.8**	92.5	38.5	91.5	41.7
**2556.3**	92.0	38.5	91.5	40.0
**2583.1**	91.4	38.5	91.4	38.5
**2656**	90.9	38.5	91.4	37.0

**2737.9**	90.9	42.3	91.9	39.3
**2754.1**	90.4	42.3	91.8	37.9
**2794.4**	90.4	46.2	92.3	40.0
**2955.4**	89.8	46.2	92.3	38.7
**3083.1**	89.3	46.2	92.3	37.5
**3102.1**	88.8	46.2	92.2	36.4
**3209.7**	88.8	50.0	92.7	38.2
**3358.9**	88.2	50.0	92.7	37.1
**3589.8**	87.7	50.0	92.7	36.1
**3771.3**	87.2	50.0	92.6	35.1
**3832.6**	86.6	50.0	92.6	34.2
**3902.8**	86.1	50.0	92.5	33.3
**3961.2**	85.6	50.0	92.5	32.5
**4088.2**	85.0	50.0	92.4	31.7
**4490**	84.5	50.0	92.4	31.0
**4818.5**	84.0	50.0	92.4	30.2
**4935**	83.4	50.0	92.3	29.5
**5094.4**	83.4	53.8	92.9	31.1
**5169.9**	82.9	53.8	92.8	30.4
**5238.4**	82.4	53.8	92.8	29.8
**5312.8**	81.8	53.8	92.7	29.2
**5344.2**	81.3	53.8	92.7	28.6
**5368.1**	80.7	53.8	92.6	28.0
**5425.6**	80.2	53.8	92.6	27.5
**5567.1**	79.7	53.8	92.5	26.9
**5811.8**	79.7	57.7	93.1	28.3
**6055.1**	79.1	57.7	93.1	27.8
**6161.6**	78.6	57.7	93.0	27.3
**6250**	78.1	57.7	93.0	26.8
**6354.1**	77.5	57.7	92.9	26.3
**6398.2**	77.0	57.7	92.9	25.9
**6451.7**	76.5	57.7	92.9	25.4
**6490.6**	75.9	57.7	92.8	25.0
**6503.3**	75.4	57.7	92.8	24.6
**6544.5**	74.9	57.7	92.7	24.2
**6588.5**	74.3	57.7	92.7	23.8
**6701.2**	73.8	57.7	92.6	23.4
**6885.6**	73.3	57.7	92.6	23.1
**6969.7**	72.7	57.7	92.5	22.7
**6985**	72.7	61.5	93.2	23.9
**7055**	72.2	61.5	93.1	23.5
**7158.6**	71.7	61.5	93.1	23.2
**7202.5**	71.1	61.5	93.0	22.9
**7233.1**	70.6	61.5	93.0	22.5
**7276.5**	70.1	61.5	92.9	22.2
**7348.4**	69.5	61.5	92.9	21.9
**7423.5**	69.0	61.5	92.8	21.6
**7494.7**	69.0	65.4	93.5	22.7
**7556.9**	68.4	65.4	93.4	22.4
**7704.8**	67.9	65.4	93.4	22.1
**7848.6**	67.4	65.4	93.3	21.8
**7857.1**	66.8	65.4	93.3	21.5
**7949.7**	66.3	65.4	93.2	21.2
**8071.1**	65.8	65.4	93.2	21.0
**8103.8**	65.8	69.2	93.9	22.0

**8148.6**	65.2	69.2	93.8	21.7
**8204.6**	64.7	69.2	93.8	21.4
**8217.6**	64.2	69.2	93.8	21.2
**8286.3**	63.6	69.2	93.7	20.9
**8376.2**	63.1	69.2	93.7	20.7
**8455.2**	63.1	73.1	94.4	21.6
**8700.3**	62.6	73.1	94.4	21.3
**8916.2**	62.6	76.9	95.1	22.2
**9103.8**	62.0	76.9	95.1	22.0
**9305.1**	61.5	76.9	95.0	21.7
**9439.1**	61.0	76.9	95.0	21.5
**9612.6**	60.4	76.9	95.0	21.3
**9726.5**	59.9	76.9	94.9	21.1
**9809.1**	59.4	76.9	94.9	20.8
**9875.9**	58.8	76.9	94.8	20.6
**9963.4**	58.3	76.9	94.8	20.4
**10046.8**	57.8	76.9	94.7	20.2
**10235.3**	57.2	76.9	94.7	20.0
**10519.2**	56.7	76.9	94.6	19.8
**10832.7**	56.1	76.9	94.6	19.6
**11087.8**	55.6	76.9	94.5	19.4
**11166.3**	55.1	76.9	94.5	19.2
**11265**	54.5	76.9	94.4	19.0
**11361.8**	54.5	80.8	95.3	19.8
**11433**	54.0	80.8	95.3	19.6
**11486.2**	53.5	80.8	95.2	19.4
**11501.5**	52.9	80.8	95.2	19.3
**11758.8**	52.4	80.8	95.1	19.1
**12059**	51.9	80.8	95.1	18.9
**12114**	51.3	80.8	95.0	18.8
**12281.7**	50.8	80.8	95.0	18.6
**12478.8**	50.3	80.8	94.9	18.4
**12574.1**	49.7	80.8	94.9	18.3
**12636.3**	49.2	80.8	94.8	18.1
**12672.3**	48.7	80.8	94.8	17.9
**12932.3**	48.1	80.8	94.7	17.8
**13159.7**	47.6	80.8	94.7	17.6
**13212.5**	47.1	80.8	94.6	17.5
**13292.9**	46.5	80.8	94.6	17.4
**13354.2**	46.5	84.6	95.6	18.0
**13432.5**	46.0	84.6	95.6	17.9
**13485.1**	45.5	84.6	95.5	17.7
**13543**	44.9	84.6	95.5	17.6
**13639.7**	44.4	84.6	95.4	17.5
**13700.7**	43.9	84.6	95.3	17.3
**13878**	43.3	84.6	95.3	17.2
**14099.3**	42.8	84.6	95.2	17.1
**14184.2**	42.2	84.6	95.2	16.9
**14584.6**	41.7	84.6	95.1	16.8
**14992.7**	41.2	84.6	95.1	16.7
**15118.9**	41.2	88.5	96.2	17.3
**15390.9**	40.6	88.5	96.2	17.2
**15586**	40.1	88.5	96.2	17.0
**15687.3**	39.6	88.5	96.1	16.9
**15773.7**	39.0	88.5	96.1	16.8

**15837.8**	38.5	88.5	96.0	16.7
**15929.4**	38.0	88.5	95.9	16.5
**16109.8**	37.4	88.5	95.9	16.4
**16330.6**	36.9	88.5	95.8	16.3
**16404.8**	36.4	88.5	95.8	16.2
**16417.2**	35.8	88.5	95.7	16.1
**16513**	35.3	88.5	95.7	16.0
**16617.4**	34.8	88.5	95.6	15.9
**16930.7**	34.2	88.5	95.5	15.8
**17243.3**	33.7	88.5	95.5	15.6
**17566.1**	33.2	88.5	95.4	15.5
**17895.5**	32.6	88.5	95.3	15.4
**18177.6**	32.1	88.5	95.2	15.3
**18713.3**	31.6	88.5	95.2	15.2
**19051.1**	31.0	88.5	95.1	15.1
**19316.6**	30.5	88.5	95.0	15.0
**20078.4**	29.9	88.5	94.9	14.9
**20651.8**	29.4	88.5	94.8	14.8
**20822**	28.9	88.5	94.7	14.7
**21037.4**	28.3	88.5	94.6	14.6
**21186.7**	27.8	88.5	94.5	14.6
**21308.7**	27.3	88.5	94.4	14.5
**21377.7**	26.7	88.5	94.3	14.4
**21808**	26.7	92.3	96.2	14.9
**22636.4**	26.2	92.3	96.1	14.8
**23121.1**	25.7	92.3	96.0	14.7
**23213.5**	25.1	92.3	95.9	14.6
**23306.7**	24.6	92.3	95.8	14.5
**23496.2**	24.1	92.3	95.7	14.5
**23691.8**	23.5	92.3	95.7	14.4
**23936.7**	23.0	92.3	95.6	14.3
**24255.2**	22.5	92.3	95.5	14.2
**24474.1**	21.9	92.3	95.3	14.1
**24984**	21.4	92.3	95.2	14.0
**25615.7**	20.9	92.3	95.1	14.0
**26023.4**	20.3	92.3	95.0	13.9
**26332.6**	19.8	92.3	94.9	13.8
**26898.7**	19.3	92.3	94.7	13.7
**27569.2**	18.7	92.3	94.6	13.6
**28334.2**	18.2	92.3	94.4	13.6
**30052.9**	17.6	92.3	94.3	13.5
**31235.4**	17.1	92.3	94.1	13.4
**31294.4**	16.6	92.3	93.9	13.3
**31458.9**	16.0	92.3	93.8	13.3
**31605.6**	15.5	92.3	93.5	13.2
**31813.5**	15.0	92.3	93.3	13.1
**32204.9**	14.4	92.3	93.1	13.0
**32687.5**	13.9	92.3	92.9	13.0
**33385.1**	13.4	92.3	92.6	12.9
**33944.7**	12.8	92.3	92.3	12.8
**34822.2**	12.8	96.2	96.0	13.3
**36188.2**	12.3	96.2	95.8	13.2
**36832.2**	11.8	96.2	95.7	13.2
**37176.2**	11.2	96.2	95.5	13.1
**38803.6**	10.7	96.2	95.2	13.0

**40623.4**	10.7	100.0	100.0	13.5
**42406.4**	10.2	100.0	100.0	13.4
**44296.6**	9.6	100.0	100.0	13.3
**44955.6**	9.1	100.0	100.0	13.3
**45371.9**	8.6	100.0	100.0	13.2
**46514.1**	8.0	100.0	100.0	13.1
**47291.5**	7.5	100.0	100.0	13.1
**47758.7**	7.0	100.0	100.0	13.0
**48550.6**	6.4	100.0	100.0	12.9
**48957.3**	5.9	100.0	100.0	12.9
**49031.7**	5.3	100.0	100.0	12.8
**49223.8**	4.8	100.0	100.0	12.7
**49467.3**	4.3	100.0	100.0	12.7
**49644.6**	3.7	100.0	100.0	12.6
**51048.6**	3.2	100.0	100.0	12.6
**53869**	2.7	100.0	100.0	12.5
**58003.7**	2.1	100.0	100.0	12.4
**61299.1**	1.6	100.0	100.0	12.4
**64766.9**	1.1	100.0	100.0	12.3
**214908.8**	0.5	100.0	100.0	12.3

Table S3: Association of Variables with Success in Each Passage:

Continuous quantitative variables are described with the median and interquartile ranges and are compared with the Kruskal-Wallis test. Qualitative variables are described with frequencies and percentages, and are compared with the Chi-square test or Fisher's exact test.

Variable	PX1	PX2	PX3	P
Age	69.0 (14.0)	62.5 (20.0)	64.0 (17.0)	0.656
Lymphocytes	1500 (1400)	1550 (1025)	1400 (1000)	0.570
Platelets	256000 (87000)	250000 (106000)	256000 (101000)	0.923
Neutrophils	4400 (2850)	4600 (3175)	4800 (3500)	0.690
LDH	311 (178)	286 (182)	347 (188)	0.047
NLR	2.79 (3.79)	3.19 (2.92)	3.29 (4.35)	0.503
PLR	172 (159)	186 (156)	182 (154)	0.919
NLR				0.710
<=5	33 (70.2%)	20 (76.9%)	129 (69.0%)	
>5	14 (29.8%)	6 (23.1%)	58 (31.0%)	
LDH				0.107
≤250	18 (38.3%)	10 (38.5%)	47 (25.1%)	
>250	29 (61.7%)	16 (61.5%)	140 (74.9%)	
Platelets				0.599
≤150000	3 (6.4%)	0 (0.0%)	13 (7.0%)	
>150000	44 (93.6%)	26 (100.0%)	174 (93.0%)	
Lymphocytes				0.825
≤1000	13 (27.7%)	6 (23.1%)	54 (28.9%)	
>1000	34 (72.3%)	20 (76.9%)	133 (71.1%)	
Neutrophils				0.457
≤1500	0 (0.0%)	1 (3.8%)	3 (1.6%)	
>1500	47 (100.0%)	25 (96.2%)	184 (98.4%)	
PLR2				0.392
≤138	16 (34.0%)	10 (38.5%)	52 (27.8%)	
139-232	14 (29.8%)	6 (23.1%)	75 (40.1%)	
>232	17 (36.2%)	10 (38.5%)	60 (32.1%)	
PLR3				0.816
≤114	13 (27.7%)	9 (34.6%)	43 (23.0%)	
115-182	12 (25.5%)	4 (15.4%)	51 (27.3%)	
183-273	11 (23.4%)	7 (26.9%)	44 (23.5%)	
>273	11 (23.4%)	6 (23.1%)	49 (26.2%)	
Sex				0.511
Male	16 (34.0%)	12 (46.2%)	79 (42.2%)	
Female	31 (66.0%)	14 (53.8%)	108 (57.8%)	
Menopausal				0.019
Premenopausal	7 (20.6%)	5 (33.3%)	10 (9.5%)	
Postmenopausal	27 (79.4%)	10 (66.7%)	95 (90.5%)	
Diabetic				0.958
No	40 (85.1%)	22 (84.6%)	155 (82.9%)	
Yes	7 (14.9%)	4 (15.4%)	32 (17.1%)	
Metformin				0.990
No	40 (85.1%)	22 (84.6%)	160 (85.6%)	
Yes	7 (14.9%)	4 (15.4%)	27 (14.4%)	
smoking habt				0.161
Never	31 (67.4%)	16 (61.5%)	97 (51.9%)	
IPA<20	7 (15.2%)	3 (11.5%)	51 (27.3%)	
IPA>20	8 (17.4%)	7 (26.9%)	39 (20.9%)	
Previous biologic				0.349

No	45 (95.7%)	23 (88.5%)	165 (88.2%)	
Yes	2 (4.3%)	3 (11.5%)	22 (11.8%)	
Previous radiotherapy				1.000
No	44 (93.6%)	24 (92.3%)	173 (92.5%)	
Yes	3 (6.4%)	2 (7.7%)	14 (7.5%)	
Antibiotics				0.314
No	32 (68.1%)	13 (50.0%)	115 (61.5%)	
Yes	15 (31.9%)	13 (50.0%)	72 (38.5%)	
Steroids				0.455
No	42 (89.4%)	22 (84.6%)	153 (81.8%)	
Yes	5 (10.6%)	4 (15.4%)	34 (18.2%)	
Grade				0.747
Well differentiated	15 (31.9%)	5 (20.0%)	47 (25.7%)	
Moderately differentiated	16 (34.0%)	11 (44.0%)	63 (34.4%)	
Poorly differentiated	16 (34.0%)	9 (36.0%)	73 (39.9%)	
Lymphovascular invasion				0.209
Nil	28 (59.6%)	16 (61.5%)	90 (48.1%)	
Present	19 (40.4%)	10 (38.5%)	97 (51.9%)	
Neural invasion				0.507
Nil	35 (74.5%)	20 (76.9%)	126 (68.1%)	
Present	12 (25.5%)	6 (23.1%)	59 (31.9%)	
KRAS				0.520
Wild type	6 (85.7%)	2 (50.0%)	32 (65.3%)	
Mutant	1 (14.3%)	2 (50.0%)	17 (34.7%)	
NRAS				0.510
Wild type	5 (83.3%)	2 (100.0%)	30 (93.8%)	
Mutant	1 (16.7%)	0 (0.0%)	2 (6.2%)	
BRAF				1.000
Wild type	7 (100.0%)	2 (100.0%)	52 (92.9%)	
Mutant	0 (0.0%)	0 (0.0%)	4 (7.1%)	
Oncopanel Yes/No				0.251
No	43 (91.5%)	25 (96.2%)	181 (96.8%)	
Yes	4 (8.5%)	1 (3.8%)	6 (3.2%)	
dMMR				0.151
Proficient	16 (100.0%)	8 (88.9%)	75 (80.6%)	
Deficient	0 (0.0%)	1 (11.1%)	18 (19.4%)	
MSI				0.254
MSS	0 (0.0%)	0 (0.0%)	11 (39.3%)	
MSI-H	0 (0.0%)	1 (100.0%)	14 (50.0%)	
MSI-L	1 (100.0%)	0 (0.0%)	3 (10.7%)	
HER2				0.546
0	6 (12.8%)	2 (7.7%)	24 (12.8%)	
1	1 (2.1%)	0 (0.0%)	0 (0.0%)	
2	40 (85.1%)	24 (92.3%)	163 (87.2%)	
BRCA				0.236
Wild type	5 (83.3%)	2 (50.0%)	22 (84.6%)	
Mutant Germline	1 (16.7%)	2 (50.0%)	4 (15.4%)	
Mutant Somatic	0 (0.0%)	0 (0.0%)	0 (0.0%)	

Table S4: Correlation of variables with Tumor Growth Rates in the first passage (PX1)

Variable	N	Correlation or median	P
Age	260	0.08	0.184
Lymphocytes	260	-0.05	0.411
Platelets	260	0.07	0.282
Neutrophils	260	0.04	0.532
LDH	260	0.01	0.883
NLR	260	0.05	0.404
PLR	260	0.06	0.299
NLR2			0.511
<=5	182	240 (428)	
>5	78	225 (473)	
LDH2			0.267
≤250	75	270 (517)	
>250	185	219 (412)	
Platelets2			0.382
≤150000	16	373 (315)	
>150000	244	230 (455)	
Lymphocytes2			0.355
<=1000	73	261 (455)	
>1000	187	229 (437)	
Neutrophils2			0.599
≤1500	4	601 (1110)	
>1500	256	237 (430)	
PLR2			0.516
≤138	78	196 (408)	
139-232	95	258 (394)	
>232	87	255 (590)	
PLR3			0.360
≤114	65	196 (415)	
115-182	67	184 (467)	
183-273	62	270 (468)	
>273	66	258 (505)	
Sex			0.667
Male	107	263 (352)	
Female	153	210 (509)	
Menopausal			0.031
Premenopausal	22	116 (294)	
Postmenopausal	132	231 (516)	
Diabetic			0.398
No	217	239 (426)	
Yes	43	198 (536)	
Metformin			0.285
No	222	237 (419)	
Yes	38	229 (567)	
smoking habt			0.414
Never	144	214 (489)	
IPA<20	61	281 (447)	
IPA>20	54	247 (341)	
Previous biologic			0.018
No	233	210 (421)	
Yes	27	363 (619)	
Previous radiotherapy			0.849
No	241	239 (441)	

Yes	19	200 (500)	
Antibiotics			0.760
No	160	233 (400)	
Yes	100	240 (508)	
Steroids			0.092
No	217	230 (449)	
Yes	43	264 (522)	
Grade			0.144
Well differentiated	67	289 (540)	
Moderately differentiated	90	172 (438)	
Poorly differentiated	98	233 (408)	
Lymphovascular invasion			0.775
Nil	134	214 (416)	
Present	126	264 (462)	
Neural invasion			0.014
Nil	181	200 (423)	
Present	77	319 (534)	
KRAS			0.907
Wild type	40	278 (659)	
Mutant	20	398 (491)	
NRAS			0.159
Wild type	37	426 (697)	
Mutant	3	50.9 (329)	
BRAF			0.045
Wild type	61	360 (611)	
Mutant	4	785 (620)	
Oncopanel Yes/No			0.583
No	249	239 (431)	
Yes	11	103 (780)	
dMMR			0.838
Proficient	99	344 (607)	
Deficient	19	431 (295)	
MSI			0.091
MSS	11	184 (277)	
MSI-H	15	451 (312)	
MSI-L	4	228 (202)	
HER2			0.235
0	32	264 (554)	
1	1	-92	
2	225	231 (427)	
3	2	752 (95.5)	
BRCA			0.206
Wild type	29	235 (641)	
Mutant Germline	7	22.7 (132)	
Mutant Somatic	0		
N mice implanted			0.584
1	77	277 (460)	
2	58	174 (532)	
3	54	250 (388)	
4	33	200 (373)	
5	32	149 (434)	

Table S5: Correlation of variables with Tumor growth Rates in the second passage (PX2).

Variable	N	Correlation or median	P
Age	213	0.06	0.355
Lymphocytes	213	0.02	0.732
Platelets	213	0.11	0.102
Neutrophils	213	0.1	0.145
LDH	213	0.03	0.696
NLR	213	0.06	0.389
PLR	213	0.06	0.417
NLR2			0.552
<=5	149	11335 (15886)	
>5	64	12377 (15411)	
LDH2			0.736
≤250	57	10411 (17050)	
>250	156	11433 (15765)	
Platelets2			0.308
≤150000	13	15602 (19103)	
>150000	200	11166 (15411)	
Lymphocytes2			0.482
<=1000	60	13160 (14899)	
>1000	153	10627 (15814)	
Neutrophils2			0.828
≤1500	4	14306 (15880)	
>1500	209	11389 (15325)	
PLR2			0.250
≤138	62	11336 (19236)	
139-232	81	9263 (11869)	
>232	70	13371 (16686)	
PLR3			0.184
≤114	52	11960 (18553)	
115-182	55	7547 (14253)	
183-273	51	12108 (13100)	
>273	55	13478 (16390)	
Sex			0.171
Male	91	12120 (16158)	
Female	122	9963 (13749)	
Menopausal			0.114
Premenopausal	15	5673 (10316)	
Postmenopausal	105	9759 (15751)	
Diabetic			0.137
No	177	11038 (15708)	
Yes	36	13773 (16859)	
Metformin			0.144
No	182	11088 (15824)	
Yes	31	14159 (14626)	
smoking habt			0.142
Never	113	9347 (14942)	
IPA<20	54	13640 (14752)	
IPA>20	46	9976 (15194)	
Previous biologic			0.002
No	188	10833 (12669)	
Yes	25	23634 (33098)	
Previous radiotherapy			0.449
No	197	11335 (16024)	

Yes	16	13211 (13430)	
Antibiotics			0.613
No	128	10627 (16009)	
Yes	85	12108 (14797)	
Steroids			0.108
No	175	10059 (15454)	
Yes	38	13400 (18988)	
Grade			0.603
Well differentiated	52	11336 (14104)	
Moderately differentiated	74	11362 (15943)	
Poorly differentiated	82	11753 (16201)	
Lymphovascular invasion			0.568
Nil	106	9777 (15597)	
Present	107	12444 (14857)	
Neural invasion			0.104
Nil	146	9959 (12950)	
Present	65	13161 (20019)	
KRAS			0.833
Wild type	34	16617 (21870)	
Mutant	19	15602 (33867)	
NRAS			0.471
Wild type	32	21030 (21280)	
Mutant	2	12965 (3434)	
BRAF			0.024
Wild type	54	16331 (22786)	
Mutant	4	46112 (8075)	
Oncopanel Yes/No			0.958
No	206	11433 (15227)	
Yes	7	9694 (26084)	
dMMR			0.884
Proficient	83	15570 (20629)	
Deficient	19	13716 (31507)	
MSI			0.213
MSS	11	10059 (20484)	
MSI-H	15	16425 (38455)	
MSI-L	3	5951 (2238)	
HER2			0.818
0	26	13737 (20321)	
1	0	NaN	
2	185	11195 (15636)	
3	2	18985 (12648)	
BRCA			0.210
Wild type	24	9718 (20579)	
Mutant Germline	6	4968 (3953)	
Mutant Somatic	0		

Table S6: Correlation of variables with Tumor Growth Rates in the third passage (PX3):

Variable	N	Correlation or median	P
Age	187	0.03	0.677
Lymphocytes	187	0.06	0.390
Platelets	187	0.1	0.168
Neutrophils	187	0.05	0.515
LDH	187	-0.09	0.234
NLR	187	0	0.962
PLR	187	0.01	0.878
NLR2			0.355
<=5	129	14141 (17259)	
>5	58	11315 (16316)	
LDH			0.009
≤250	47	18123 (18986)	
>250	140	11750 (13463)	
Platelets			0.972
≤150000	13	14892 (13322)	
>150000	174	12913 (17282)	
Lymphocytes			0.843
<=1000	54	13530 (18101)	
>1000	133	12806 (16395)	
Neutrophils			0.961
≤1500	3	19973 (10280)	
>1500	184	12913 (17400)	
PLR			0.462
≤138	52	14486 (18546)	
139-232	75	11775 (13397)	
>232	60	13530 (20146)	
PLR			0.117
≤114	43	14892 (17498)	
115-182	51	10500 (13072)	
183-273	44	13679 (17734)	
>273	49	17003 (19111)	
Sex			0.578
Male	79	14081 (14760)	
Female	108	12386 (18015)	
Menopausal			0.666
Premenopausal	10	14361 (7330)	
Postmenopausal	95	11797 (21403)	
Diabetic			0.645
No	155	12806 (17293)	
Yes	32	15273 (16861)	
Metformin			0.334
No	160	12558 (17341)	
Yes	27	16703 (15125)	
smoking habt			0.981
Never	97	11982 (18444)	
IPA<20	51	15916 (14602)	
IPA>20	39	11775 (18860)	
Previous biologic			0.000
No	165	11769 (15007)	
Yes	22	27038 (18359)	
Previous radiotherapy			0.413

No	173	12326 (16502)	
Yes	14	17456 (15415)	
Antibiotics			0.650
No	115	13927 (20113)	
Yes	72	12905 (13008)	
Steroids			0.770
No	153	12806 (16609)	
Yes	34	13530 (16462)	
Grade			0.389
Well differentiated	47	12326 (17856)	
Moderately differentiated	63	16496 (17305)	
Poorly differentiated	73	11850 (13951)	
Lymphovascular invasion			0.515
Nil	90	13086 (19756)	
Present	97	13019 (14819)	
Neural invasion			0.685
Nil	126	14004 (17511)	
Present	59	11982 (14739)	
KRAS			0.354
Wild type	32	11889 (18600)	
Mutant	17	18986 (23667)	
NRAS			0.327
Wild type	30	16572 (24312)	
Mutant	2	9545 (8578)	
BRAF			0.101
Wild type	52	14368 (18710)	
Mutant	4	34441 (17200)	
Oncopanel Yes/No			0.473
No	181	13019 (16564)	
Yes	6	19310 (22959)	
dMMR			0.789
Proficient	75	16223 (19455)	
Deficient	18	17252 (17966)	
MSI			0.073
MSS	11	5779 (11696)	
MSI-H	14	17964 (12224)	
MSI-L	3	5770 (474)	
HER2			0.949
0	24	12394 (14311)	
1	0	NaN	
2	161	13216 (17010)	
3	2	17846 (8398)	
BRCA			0.130
Wild type	22	11889 (15254)	
Mutant Germline	4	4413 (7274)	
Mutant Somatic	0		
